# Life at the Frozen Limit: Microbial Carbon Metabolism Across a Late Pleistocene Permafrost Chronosequence

**DOI:** 10.3389/fmicb.2020.01753

**Published:** 2020-07-29

**Authors:** Mary-Cathrine Leewis, Renaud Berlemont, David C. Podgorski, Archana Srinivas, Phoebe Zito, Robert G. M. Spencer, Jack McFarland, Thomas A. Douglas, Christopher H. Conaway, Mark Waldrop, Rachel Mackelprang

**Affiliations:** ^1^U.S. Geological Survey, Geology, Minerals, Energy, and Geophysics Science Center, Menlo Park, CA, United States; ^2^Department of Biological Sciences, California State University Long Beach, Long Beach, CA, United States; ^3^Pontchartrain Institute for Environmental Sciences, Department of Chemistry, University of New Orleans, New Orleans, LA, United States; ^4^Department of Biology, California State University Northridge, Northridge, CA, United States; ^5^National High Magnetic Field Laboratory Geochemistry Group, Department of Earth, Ocean, and Atmospheric Science, Florida State University, Tallahassee, FL, United States; ^6^U.S. Army Cold Regions Research and Engineering Laboratory, Fort Wainwright, AK, United States; ^7^U.S. Geological Survey, Water Resources Mission Area, Menlo Park, CA, United States

**Keywords:** permafrost, Pleistocene, carbohydrate active enzymes, CAZyme, carbon, FT-ICR MS, metagenomics

## Abstract

Permafrost is an extreme habitat yet it hosts microbial populations that remain active over millennia. Using permafrost collected from a Pleistocene chronosequence (19 to 33 ka), we hypothesized that the functional genetic potential of microbial communities in permafrost would reflect microbial strategies to metabolize permafrost soluble organic matter (OM) *in situ* over geologic time. We also hypothesized that changes in the metagenome across the chronosequence would correlate with shifts in carbon chemistry, permafrost age, and paleoclimate at the time of permafrost formation. We combined high-resolution characterization of water-soluble OM by Fourier-transform ion-cyclotron-resonance mass spectrometry (FT-ICR MS), quantification of organic anions in permafrost water extracts, and metagenomic sequencing to better understand the relationships between the molecular-level composition of potentially bioavailable OM, the microbial community, and permafrost age. Both age and paleoclimate had marked effects on both the molecular composition of dissolved OM and the microbial community. The relative abundance of genes associated with hydrogenotrophic methanogenesis, carbohydrate active enzyme families, nominal oxidation state of carbon (NOSC), and number of identifiable molecular formulae significantly decreased with increasing age. In contrast, genes associated with fermentation of short chain fatty acids (SCFAs), the concentration of SCFAs and ammonium all significantly increased with age. We present a conceptual model of microbial metabolism in permafrost based on fermentation of OM and the buildup of organic acids that helps to explain the unique chemistry of ancient permafrost soils. These findings imply long-term *in situ* microbial turnover of ancient permafrost OM and that this pooled biolabile OM could prime ancient permafrost soils for a larger and more rapid microbial response to thaw compared to younger permafrost soils.

## Introduction

Permafrost underlies one-quarter of the Northern Hemisphere and contains almost half of the Earth’s soil carbon (C) (McGuire et al., 2018). Permafrost C has accumulated over millennia through wind-blown deep loess deposits, peat development, and alluvial sedimentation (Schirrmeister et al., 2016; Brouchkov et al., 2017). Yedoma permafrost, a type of ice- and organic-rich permafrost common in continental climate zones, persists from the Pleistocene and contains up to 466 Pg of organic carbon (OC), approximately 35% of the northern permafrost C pool (Strauss et al., 2017). The high ice content places yedoma at high risk for thaw degradation in a warmer future (Brown et al., 2015). The chemical composition of permafrost C varies widely (Hodgkins et al., 2016; Schirrmeister et al., 2016; Wickland et al., 2018), and reflects the historic climate, vegetation, and timing of formation. For example, the cold DuVanny Yar Interval of the late Pleistocene (approximately 25 ka) caused gradual organic matter (OM) accumulation over long periods of steady aeolian deposition which likely froze within decades to centuries after burial (Hopkins, 1982; Guthrie and Stoker, 1990; Lenz et al., 2016; Strauss et al., 2017). In contrast, warmer conditions prevalent in Beringia during the Holocene and portions of the late Pleistocene (e.g., 19–22, 30–38 ka) may have prevented accumulated OM from freezing, thus subjecting it to decomposition by microorganisms for centuries to millennia prior to permafrost formation (Hopkins, 1982; Hamilton et al., 1988; Jones et al., 2012; Kanevskiy et al., 2014). Microorganisms contained within this permafrost may be reflective of the type of organic material present but may also have the capacity to alter its chemical characteristics over time (Heslop et al., 2019).

Anaerobic decomposition within intact permafrost occurs through a series of cascading hydrolytic and fermentative steps resulting in production of CO_2_ or CH_4_ (Tveit et al., 2015; Liang et al., 2019). The first steps in degradation of plant structural polymers (e.g., cellulose, hemicellulose) are mediated by hydrolytic microorganisms which degrade these complex organic macromolecules to more simple monomeric compounds (e.g., oligosaccharides), which then can serve as substrates for primary fermentation (Kotsyurbenko, 2005; Ye et al., 2014). In cold environments primary fermentation may produce hydrogen, alcohols, and short chain fatty acids (SCFAs; e.g., acetate, formate, butyrate, propionate). Secondary fermentation then metabolizes these SCFAs to form other SCFAs (including acetate) and hydrogen by syntrophic bacteria (Tveit et al., 2015). Hydrogen and acetate are then consumed to form CO_2_ or CH_4_ in a terminal step of fermentation. This final step is necessary to create the thermodynamically favorable conditions necessary for each of the preceding fermentative steps (Panikov, 1999). Accumulation of intermediate SCFAs is thus indicative of decoupling between primary and secondary fermentative processes and the terminal fermentative steps (Duddleston et al., 2002; Hines et al., 2008). Dissolved organic matter (DOM) acts as a reservoir for the products of microbial metabolism and fermentation, thus the molecular composition of DOM can provide insight into microbial degradative pathways (Hodgkins et al., 2016).

Liquid water can exist in permafrost in nanometer-thin brine channels surrounding soil particles. Multiple factors may affect the amount of liquid water in permafrost, including temperature, pore size distribution, and solute concentration (Gilichinsky and Rivkina, 2011). Liquid water in permafrost provides a potential source of OC and nutrients to microbial cells (Jansson and Taş, 2014). Recent research demonstrates long-term *in situ* production of biolabile DOC in the unfrozen water fraction of Pleistocene permafrost (Ewing et al., 2015a). Production of DOC and build-up of SCFAs in permafrost implies microorganisms metabolize Pleistocene-aged DOC in the liquid water phase of yedoma despite the absence of new inputs of organic macromolecules and nutrients (Drake et al., 2015). However, accumulation of these SCFAs may also indicate a breakdown in syntrophic bacterial metabolism as the system becomes thermodynamically limited due to pooling of end products (Jackson and McInerney, 2002).

Permafrost soils host a diverse microbial community, though diversity is typically reduced relative to active layer soils, pointing to its unique environmental stressors (e.g., reduction in energy availability and salinity) (Mackelprang et al., 2011; Hultman et al., 2015; Rivkina et al., 2016). We still do not understand the mechanisms that enable microbial survival and growth in permafrost nor how microbial communities respond to increasingly thermodynamically limited conditions given the closed nature of the system (Rivkina et al., 2000; Tuorto et al., 2014; Hultman et al., 2015; Burkert et al., 2019). The overall understanding of C cycling-related metabolic processes that occur below 0°C may be inferred from genetic changes in the microbial community and the distribution of functional traits (Mackelprang et al., 2011; Hultman et al., 2015). By linking DOC quality and molecular composition of intact permafrost OM with microbial C-processing genes it may be possible to reconstruct the pathways used for microbial OM metabolism which enable survival across millennia or longer (Tveit et al., 2013, 2015; Bottos et al., 2018; Burkert et al., 2019; Liang et al., 2019).

Here we examine the relationships between the molecular composition of DOM and the functional potential of microbial communities across a Pleistocene-aged (19 to 33 ka) permafrost chronosequence. We sought to determine if changes in DOM chemistry were consistent with microbial community changes and biochemical adaptations inferred from metagenomic data. With these data, we identified cryogenic and energetic constraints on microbial metabolism of OM. Results from this study will help develop our understanding of biological activity in intact permafrost, as well as dominant microbial and ecosystem responses to permafrost thaw.

## MATERIALS AND METHODS

### Sample Collection, DNA Extraction, and Radiocarbon Dating

Permafrost cores were collected in 2012 from the Fox Permafrost Tunnel, operated by the Cold Regions Research and Engineering Laboratory (CRREL) in Fairbanks, Alaska (64.951°N – 147.621°W), and are the same cores used by Mackelprang et al. (2017). In brief, we sampled ice-rich syngenetic yedoma permafrost material corresponding to 19 ka (20 m from the tunnel portal), 27 ka (54 m) and 33 ka (81 m) before present (Supplementary Figure 1). Before coring, the sampling surface was scraped to remove a sublimated silt layer to expose fresh material. Using a sterilized round 10 cm diameter hole saw attached to a power drill, four replicate cores from each permafrost age category were collected, approximately 10–20 cm apart. To test for contamination prior to DNA extraction, each core was sprayed with 0.5 μm Fluoresbrite Yellow Green Microspheres (Polyscience, Warrington, PA, United States). The entire surface was removed using autoclaved knives and chisels, and the interior sections to be used for DNA extraction were viewed under a microscope to verify there was no surface contamination. Once cleaned from potential surface contamination, DNA was extracted from 0.5 g of soil using the FastDNA SPIN Kit for Soil (MP Biomedicals, Santa Ana, CA, United States) according to the manufacturer’s protocol, with the addition of a clean-up and purification step using the Power-Clean DNA Clean-Up Kit (MoBio Laboratories, Carlsbad, CA, United States). Calendar ages were estimated by radiocarbon dating of both soil CO_2_ and bulk soil organic matter then converting to calendar years, for details see Mackelprang et al. (2017). The youngest samples, herein referred to as “19 ka,” were dated to between 16 and 22 ka, the intermediate samples (“27 ka”) were dated to between 26 and 27 ka, and the oldest samples (“33 ka”) were dated between 32 and 33 ka. It should be noted that although there are exposures of thaw features in the Tunnel, these are limited to small subsurface hydrologic erosion events and none of the sites sampled herein exhibit any post-depositional signs of thaw degradation (Douglas et al., 2011; Douglas and Mellon, 2019). Temperatures in the tunnel have remained below freezing (approximately −3°C) since excavation was initiated in the mid-1960s (Douglas and Mellon, 2019).

### Metagenomic Sequencing and Analysis

A portion of the metagenomic data presented here was previously published in a paper focusing on microbial survival mechanisms (Mackelprang et al., 2017), all other data and analyses are unique to this article. The sequence data used here can be found in the NCBI short read archive under accession number SRP093781. Briefly, libraries from 12 samples (4 replicates per age category) were constructed using emulsion PCR (Williams et al., 2006; Mackelprang et al., 2017). We performed 2 × 100 paired end shotgun sequencing on an Illumina HiSeq instrument generating approximately 264 Gb of high-quality sequence data. Reads were quality filtered and trimmed using the FastX toolkit^1^, for further details on sequence processing please see Mackelprang et al., 2017.

To identify and annotate reads from genes potentially involved in polysaccharide processing (CAZymes) we performed a two-step process where (1) potential carbohydrate-utilization reads were identified through a blastx-like comparison to dbCAN using permissive parameters and (2) carbohydrate-active enzyme reads were further identified and annotated through comparison to a custom database of HMM profiles using an *e*-value cutoff of < 10^–5^ (Talamantes et al., 2016). For the first step, we compared raw reads to the dbCAN database (downloaded July 2016) (Yin et al., 2012) using DIAMOND v 0.8.17 (Buchfink et al., 2014) with a permissive *e*-value cutoff of 0.1. This two-step process was used to reduce the number of reads for the HMM profile step, thus reducing computational burden.

Reads were also annotated through comparison to the Kyoto Encyclopedia of Genes and Genomes (KEGG) database (Kanehisa et al., 2017) and details are available in Mackelprang et al. (2017). For both CAZyme and KEGG annotations, the number of reads matching KEGG orthologous groups or CAZyme families were counted and normalized by dividing the number of reads matching KEGG orthologous group or CAZyme family to the total number of reads matching each database to compare the distribution of genes across samples. All CAZyme data can be found in the USGS Science Base Data Repository at https://www.sciencebase.gov/catalog/item/5cd08fb1e4b09b8c0b79a466.

### Permafrost Filtrate Extraction

In the laboratory, the same cores used for metagenomic analyses were disaggregated and sub-sampled for chemical analyses. One portion was thawed and used for pH, δ^13^C-DOC, and gravimetric ice content (Mackelprang et al., 2017). To collect water extractable OM, approximately 50 g of frozen permafrost was crushed to a 1–5 mm aggregate consistency then it was placed into a combusted 250 mL mason jar with an equivalent amount of ultra-pure water (>18.2 MΩ cm resistivity, <4 ppb TOC, 0.2 μm final filtration). Jars were capped and the permafrost:water slurry was extracted overnight at 4°C with gentle shaking, then vacuum-extracted through a combusted type A/E glass fiber filter. Filtrates were sterile filtered through a 25 mm 0.2 μm GD/X syringe filter into acid washed polycarbonate bottles (Haney et al., 1999; Waldrop et al., 2010). Processed filtrates of water extractable OC were frozen immediately following collection. Three process blanks were collected concurrently under the same conditions. This collected filtrate was then used for the analysis of total DOC, major anions, organic acids, soluble NH_4_^+^, NO_3_^–^, NO_2_^–^, and water-soluble OM composition.

### Physiochemical Analyses

Extracted permafrost filtrates were analyzed for anions by ion chromatography using a Metrohm 881 Compact IC pro with detection by suppressed conductivity. Anion determinations were performed using a Metrosep A Supp 7/250 column with 3.6 mM Na_2_CO_3_ eluent (0.8 mL/min) at 45°C. A complementary set of determinations were performed targeting organic acid anions using a Metrosep Organic Acids 250/7.8 column with 0.5 mM H_2_SO_4_ eluent (0.5 mL/min) at 30°C.

Soluble NH_4_^+^, NO_3_^–^, and NO_2_^–^ measurements were carried out using batch-automated spectrophotometry (Aquakem 250, Thermo Fisher Scientific, United States). Ammonium was determined using the Phenate method [United States Environmental Protection Agency (U.S. EPA) method 350.1]. Nitrite was determined by diazotization with sulfanilamide and nitrate was catalytically reduced to nitrite using soluble nitrate reductase in the presence of reduced nicotinamide dinucleotide (NADH; EPA Method 353.1). All samples were analyzed in duplicate. Final DOC, DIN, and SCFA data were corrected for dilution and gravimetric water content.

### Water Soluble Organic Matter Composition Determination by Fourier-Transform Ion-Cyclotron-Resonance Mass Spectrometry (FT-ICR MS)

Water-soluble OM was extracted from the same permafrost filtrates using the solid phase method described by Dittmar et al. (2008). Briefly, each sample was acidified to pH 2 and passed through a Bond Elute PPL (Agilent Technologies) cartridges that were sequentially conditioned with methanol followed by acidified (pH 2) Milli-Q water. Residual salts were rinsed from the cartridge with acidified (pH 2) Milli-Q water prior to drying the stationary phase with a stream of N_2_. The DOC was eluted with 100% MeOH (JT Baker) to a final concentration of 50 μgC mL^–1^ and stored at 4°C prior to analysis. Four analytical replicate extracts from each age class were analyzed by direct infusion negative electrospray ionization (ESI) coupled with a custom-built FT-ICR mass spectrometer equipped with a 21 tesla superconducting magnet (Bruker Corp.) (Smith et al., 2018). Molecular formulas were assigned to signals >6σ root mean square baseline noise following established rules using the EnviroOrg©^TM^ software (version 2.0, developed by Yuri E. Corilo) (Koch et al., 2007; Mostovaya et al., 2017; Hawkes et al., 2020). Molecular formulas were then classified into the categories described in Supplementary Table 1 (Hodgkins et al., 2016). We calculated the number of unique molecular formulae in an age class by identifying those compounds only present in any of the biological or technical replicates for each age class. The number of shared compounds was calculated by identifying any molecular formulae present in biological or technical replicates in any of two or three age classes.

We calculated the nominal oxidation state of carbon (NOSC) according to (Riedel et al., 2012):

$$N\,O\,S\,C=4-\left[\frac{4\,c+h-3\,n-2\,o-2\,s}{c}\right]$$

where *c*, *h*, *n*, *o*, and *s* refer to the number of atoms per formula of carbon, hydrogen, nitrogen, oxygen, and sulfur, respectively. This measure uses the elemental stoichiometry of the molecular composition of organic matter, meaning that more reduced C compounds will have a lower (more negative) NOSC than more oxidized compounds (higher NOSC). The NOSC of individual compounds present in each sample were then averaged to give the NOSC of DOC in that sample. FT-ICR MS data can be found in the USGS Science Base Data Repository at https://www.sciencebase.gov/catalog/item/5cd08fb1e4b09b8c0b79a466.

### Statistical Analyses

All statistical analyses were performed in R version 3.5.0 (R Core Team, 2018). Permafrost DOC and chemical characteristics were tested for normality using a Shapiro-Wilk test, and normal data were analyzed using a one-way analysis of variance (ANOVA) with pair-wise comparisons (Tukey HSD) to assess significant difference among variables (*P* < 0.05) across permafrost ages. Non-normal data were analyzed using a Kruskal–Wallis and *post hoc* Welch’s test with adjustment for multiple comparisons. The effect of permafrost age on the CAZyme distribution was investigated using one-way ANOVA on each CAZyme family. We then visulatized affected (*P* < 0.01) and unaffected (*P* > 0.01) CAZyme families using a heat map based on the Bray-Curtis dissimilarity index and UPGMA clustering, all *P*-values are in Supplementary Table 2. We used the vegan package to conduct multivariate statistics including PERMANOVA, Mantel tests, multi-response permutation procedure (MRPP), and non-metric multidimensional scaling (NMDS) using the Bray-Curtis dissimilarity measure with vector fitting of associated chemical data (Table 1; Oksanen et al., 2017).

**TABLE 1:** Chemical characterization of permafrost filtrates from the Fox Tunnel Permafrost Chronosequence, different letters indicate statistical differences across ages, within a category (*P* < 0.05). For example, ice content (as % dry weight) was not significantly different between 19 and 33 ka, but 27 ka was significantly different from both 19 and 33 ka.

**Age**	**19 ka**	**27 ka**	**33 ka**
pH*	7.730.32*a*	7.750.14*a*	7.340.09*a*
Ice content**	39.7210.25*a*	121.0514.09*b*	51.755.24*a*
Chloride	649.32169.22*a*	95.797.82*b*	237.0256.24*c*
Sulfate	97.1270.48*a*	56.108.65*a*	165.4874.65*a*
NH4 +	528.4954.25*a*	556.5040.96*a*	1815.74136.01*b*
NO3	0.630.09*a*	1.371.17*a*	0.480.08*a*
NO2	0.250.14*a*	0.610.10*b*	0.260.02*a*
δ13C-DOC	−26.20.3*a*	−26.90.2*b*	−27.80.1*c*
DOC***	74.7020.10*a*	124.5025.87*a*	181.1337.15*b*
Acetate	0.910.10*a*	7.680.82*b*	40.456.89*c*
Butyrate	0.000.00*a*	3.040.57*b*	30.156.49*c*
Formate	0.380.04*a*	0.190.01*b*	0.340.04*a*
Propionate	6.001.59*a*	0.740.06*b*	4.780.85*a*
Isovalerate	1.270.36*a*	1.100.32*a*	7.311.56*b*
Glutarate	0.030.01*a*	0.010.00*b*	0.030.01*a*
Malate	0.160.03*a*	0.180.04*a*	0.260.02*b*
%C***	3.141.94*a*	3.110.26*a*	3.200.39*a*
%N***	0.270.16*a*	0.210.06*a*	0.270.03*a*
C/N***	11.811.14*a*	15.253.30*a*	12.100.98*a*

*^∗^Data other than pH, ice content, and δ13C-DOC are in micromoles per liter. DOC (dissolved organic carbon), acetate, butyrate, propionate, isovalerate, glutarate, and malate are reported in millimoles of carbon per liter. All measurements corrected for dilution during measurement. Average values reported as mean ± standard deviation. ^∗∗^% dry weight. ^∗∗∗^Data corrected from Mackelprang et al. (2017).*

## Results

### Permafrost Chemical Characteristics Across the Chronosequence

Permafrost DOC concentrations increased across the three sampled ages with %C, %N, and C:N remained remarkably consistent across the chronosequence (Table 1). Concentrations of SCFAs accounted for 13, 10, and 46% of total measured DOC on a molar basis in the 19, 27, and 33 ka samples, respectively. Acetate, butyrate, isovalerate, and malate concentrations all increased with increasing age of the permafrost (Table 1). Formate, glutarate, and propionate concentrations did not display a distinct time-associated pattern across the chronosequence. Average ammonium concentrations were higher for the oldest permafrost sample, while nitrate and nitrite concentrations were highest in the 27 ka samples, and chloride and sulfate concentrations were lowest in the 27 ka samples (Table 1). Nitrate demonstrated no consistent pattern with age due in part to high variability among 27 ka samples. The pH was near neutral and did not differ significantly among the three sample locations (Table 1; Mackelprang et al., 2017).

### Molecular Composition of DOM

**FIGURE 1:**
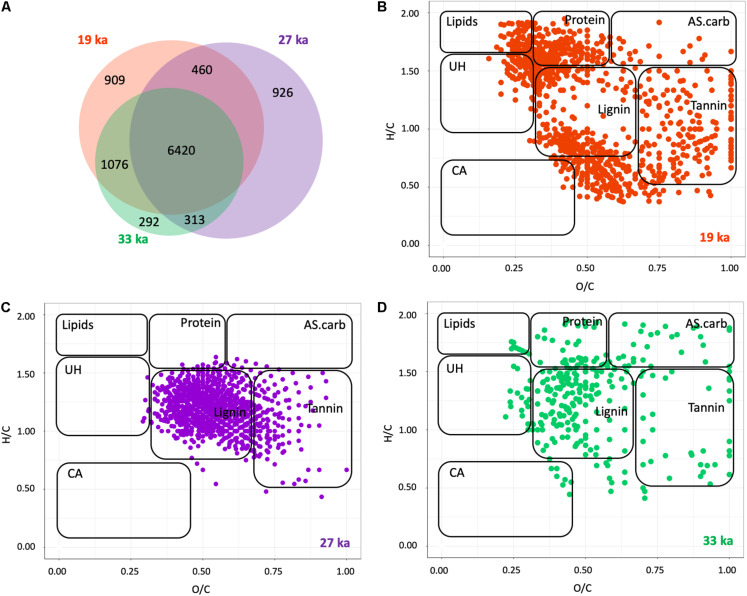
Molecular composition of DOM shifts across the permafrost chronosequence. **(A)** Venn diagram of the unique and shared number of identified molecular formulae. **(B–D)** Van Krevelen diagrams based on FT-ICR MS peaks uniquely present in each of the DOM samples from **(B)** 19 ka, **(C)** 27 ka, and **(D)** 33 ka, general compound class ranges as described in Hodgkins et al. (2016). Compound classes are defined in Table S1 and as follows: Lipid, lipid-like; Protein, protein-like; AS.carb. aminosugar and carbohydrate-like; UH, unsaturated hydrocarbons; CA, condensed aromatics; Lignin, lignin-like; Tannin, tannin-like. The relative abundance of each class is found in Supplementary Table 3.

Molecular formulae assigned to peaks detected in DOM samples from across the chronosequence totaled 10,396 (Supplementary Table 3 and Figure 1). The number of molecular formulae detected within each sample decreased with increasing permafrost age (Supplementary Table 3). Over half of the identified molecular formulae were found in all samples, with a slightly smaller number of unique formulae found in the 19 and 27 ka samples (Figure 1A). The DOM composition in the 33 ka samples had the least number of uniquely-assigned molecular formulae and shared 10% of formulae with the 19 ka, but only 3% with the 27 ka samples (Figure 1A). One-way ANOVAs revealed that the proportion of detected peaks of lipid-like, protein-like, and unsaturated hydrocarbon compounds did not vary significantly between 19 and 33 ka (Supplementary Table 3).

**FIGURE 2:**
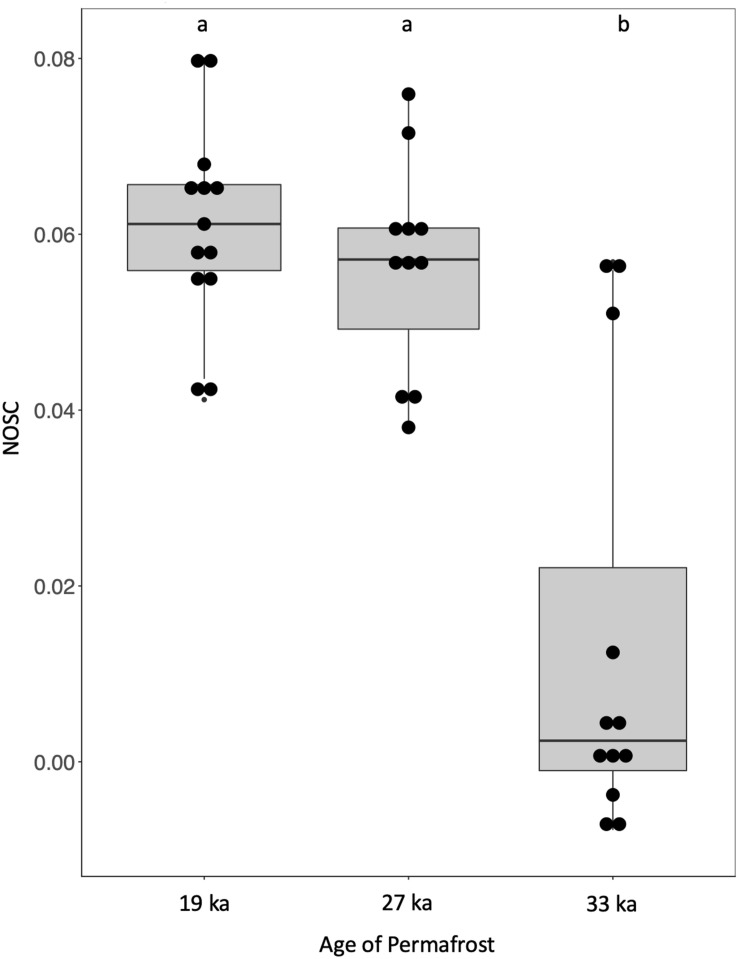
Thermodynamic favorability of DOM decreases with older samples. Nominal oxidation state of carbon (NOSC) in permafrost from across the chronosequence as calculated based on molecular formulae identified by FT-ICR MS in DOM. Different letters indicate statistically significant differences (*P* < 0.05) while vertical whisker lines indicate 95% confidence intervals (*N* = 32).

Multiple response permutation procedure (MRPP) analysis of the standardized intensities of assigned formulae indicated distinct groupings of the molecular-level composition of DOM by age, with samples from within an age class more similar to each other than to samples from other ages (Supplementary Table 4). To visualize the qualitative differences in DOM across the chronosequence, we plotted compounds unique to each age category in a van Krevelen diagram, which shows hydrogen to carbon (H:C) and oxygen to carbon (O:C) molar ratio distributions of masses (Figures 1B–D). The molecular-level composition of DOM clearly differed between samples. Lipid and protein-like compounds (H:C > 1.5) and condensed hydrocarbon-like compounds (H:C < 1) dominated the youngest samples whereas 27 ka samples demonstrated more unique lignin-like peaks at H:C 0.5–1.5. Samples from the 33 ka site, had the fewest unique peaks, which were more evenly distributed throughout van Krevelen space (Figures 1B–D). We further examined the biogeochemical reactivity of OC in each sample by calculating the NOSC, a parameter which provides information of the average oxidation state of all C formula determined by FT-ICR MS (Riedel et al., 2012). NOSC can therefore be an indirect measure of the thermodynamic favorability of C compounds available for microbial respiration, where substrates with a lower NOSC indicate lower favorability (Boye et al., 2017). The NOSC from the 33 ka samples was lower than that for younger samples; the NOSC of 19 and 27 ka samples did not significantly differ (Figure 2).

### Changes in Microbial Functional Genes With Age

**FIGURE 3:**
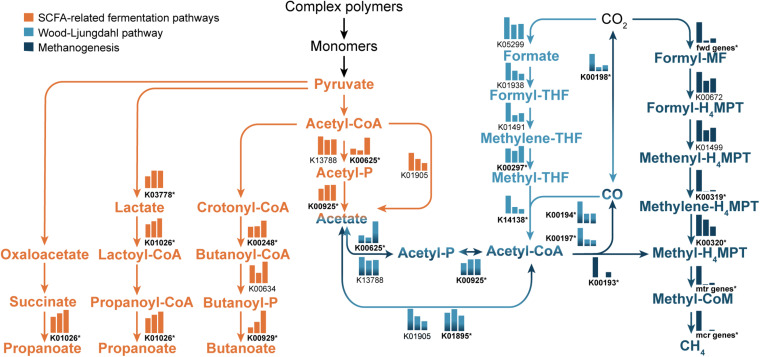
Pathways related to acetate, butanoate, and propanoate metabolism. SCFA-related fermentation pathways are shown in orange, the Wood-Ljungdahl pathway is shown in light blue, and the methanogenesis pathway is shown in dark blue. Pathway components with multiple colors indicate participation in multiple pathways. Bar charts show the relative abundance of KEGG genes in 19, 27, and 33 ka samples, respectively, from left to right on each chart. *Y*-axis values differ between genes in order to show changes in relative abundance across the chronosequence. Stars next to gene names indicate significant differences between at least one pair of age categories (i.e., 19 ka vs. 27 ka, 19 ka vs. 33 ka, or 27 ka vs. 33 ka). The bar charts for fwd, mcr, and mtr genes show the average relative abundance of multiple genes (fwd genes: K00200, K00201*, K00202*, K00203*, K00204, K00205*, K11261*, K11260*; mcr genes: K00399*, K00400*, K00402*, K03421*, K03422*, K14082; mtr genes: K00577*, K00578*, K00579*, K00580*, K00581*, K00583*, K00584). Abundance data and *p*-values for each gene are found in Supplementary Table 5).

Shotgun metagenomic sequencing resulted in an average of 22 Gb of sequence data per sample (Mackelprang et al., 2017). Globally, the functional potential within samples, predicted by annotating sequence reads against the KEGG and CAZyme databases, varied significantly between age categories (Supplementary Table 4). We next compared the functional potential of microbial SCFA metabolism between ages using a pathway-based approach (Figure 3 and Supplementary Table 5). In many cases we saw an increase in the abundance of genes associated with fermentation such as butyrate kinase (K00929), acetate kinase (K00925), and propionate CoA-transferase (K01026). Genes associated with formation of acetate via the Wood-Ljungdahl pathway generally decreased with age, with the exception of methylene-THF reductase (K00297), which increased from 19 to 33 ka. Key genes of the hydrogenotrophic methanogenesis pathway decreased drastically across the chronosequence (Figure 3). We also examined genes involved in nitrification but found no significant differences across ages (*P* > 0.9), and no evidence for anammox-associated genes.

**FIGURE 4:**
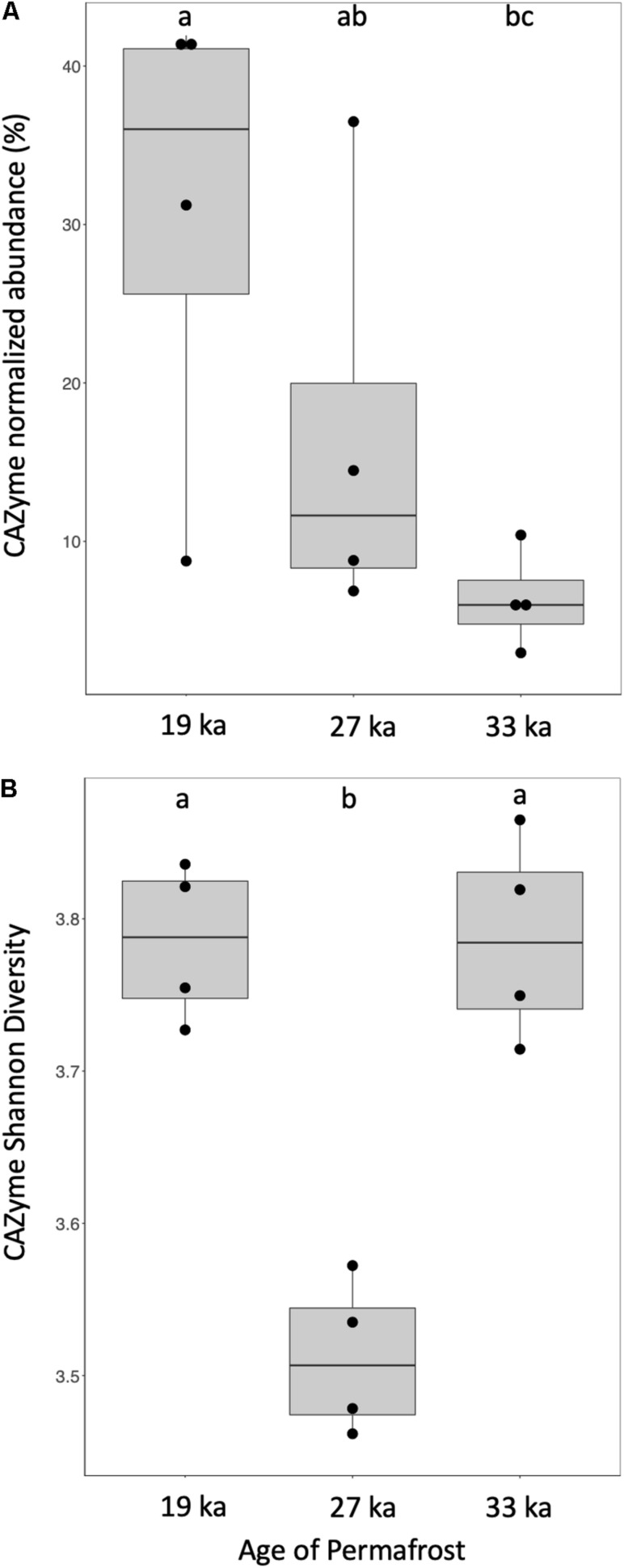
Changes in abundance and diversity of CAZymes with soil age. **(A)** The relative abundance of CAZyme PFAMs normalized to a single-copy ribosomal gene (K02874) within total identified KO’s present across the ages. **(B)** Shannon diversity measurements compared across age categories. Different letters indicate statistically significant differences (*P* < 0.05) while vertical whisker lines indicate 95% confidence intervals (*N* = 12).

Next, we explored the microbial communities’ potential for turnover of OC through identification of genes encoding CAZymes with a focus on sequences for glycoside hydrolases (GH), glycosyltransferases (GT), carbohydrate esterases (CE), polysaccharide lyases (PL), and associated carbohydrate-binding modules (CBM). When normalized to a single copy marker gene (the large subunit ribosomal protein L14, K02874) the abundance of CAZymes significantly decreased with increasing age (Figure 4A; Manor and Borenstein, 2015; Nayfach and Pollard, 2016). Normalization to other single copy marker genes (K02950, K02992, K02519) yielded similar results. MRPP analysis indicated that CAZyme profiles were more similar within age replicates than across the permafrost ages (Supplementary Table 4). Shannon diversity of CAZymes was lower in the intermediate age class (average *H* = 3.51; *P* = 0.0304 27 ka vs. 19 and 33 ka) compared to the 19 ka (average *H* = 3.78) and 33 ka sites (average *H* = 3.79; Figure 4B), which were not significantly different.

The most abundant GH families were GH65 and alpha-amylases from GH families 13 and 15, and the most abundant GT families were 1B, 51, 2_6, 20, and 4 (Supplementary Table 2). We identified sequences for polysaccharide deacetylases (PF01522) and carbohydrate esterases (PF05448) in all samples. Sequences for enzymes targeting structural polysaccharides (e.g., xylan and cellulose) were generally less abundant than those targeting smaller molecular weight compounds. Xylanolytic enzymes included members of GH families 10 and 30, whereas cellulolytic enzymes included members GH families 5, 6, 12, 44, and 48. We also identified few potential fungal cellulases from GH family 7, some potential cellulase/chitosanase from GH family 8, and some potential GH19 chitinases.

Our measurements of the relative abundance of CAZyme domains across the chronosequence identified several trends among gene families affected (*P* < 0.01) by sample age. The microbial community in the youngest samples displayed increased potential to target hemicellulose, microbial populations at 27 ka were associated with increased potential to target starch, and the microbial population in the oldest samples had have enzymes to target substrates more recalcitrant than starch (e.g., peptidoglycan, cellulose). Multiple domains including CE8, polysaccharide deacetylase, GT51, GT92, and the N-terminus domain of GH13 were significantly more abundant in the oldest vs. youngest age categories (*P* = 0.01). Conversely, few traits (including potential β-xylosidases from GH family 39 and potential polyspecific enzymes from GH family 4) were significantly greater in the youngest age category compared to the oldest. Several domains were significantly over-represented (*P* = 0.01) in the intermediate age category (Supplementary Figure 2A and Supplementary Table 2) including some glycoside hydrolases (e.g., GH1, GH13, GH15), several glycosyl-transferases (GT51, GT2_6, GT1B, GT20), and some accessory non-catalytic domains (e.g., CBM48). However, when considering all CAZymes significantly affected by sample age, the majority were less abundant in the intermediate samples compared to the oldest and/or youngest samples, this is in contrast to the overall trend of CAZyme abundance which decreased with age (Figure 4A). Examples include members of the GH families 2, 5, 10, 12, and 20 and members of the GT families 9 and 41 (Supplementary Figure 2A). Next, samples from 19 ka were systematically enriched in sequences for enzymes targeting xylan (i.e., CE1, GH10), GT4, GT13, GH42, and Pectate lyase whereas 33 ka-samples were enriched in potential cellulases from GH families 5, 12, 48, amongst others. Finally, many traits including potential cellulases from GH families 7 (fungal), 8, and 9, potential xylanases from GH family 11, and potential chitinases (and lysozyme) from GH families 18, 19, and 25 did not display significant variation with age (Supplementary Figure 2A). Thus, although the frequency of some CAZyme families remained stable across the chronosequence, many traits involved in carbohydrate processing displayed significant fluctuation across the chronosequence (Supplementary Figure 2B).

### Correlation Between Functional Genes and Permafrost Chemistry

We examined the relationship between the molecular composition of DOM (i.e., FT-ICR MS data as relative abundance of molecular formulae), the functional potential of the associated microorganisms (i.e., KEGG ortholog data, KOs), and the potential for polysaccharide processing (CAZyme data) by performing Mantel correlation tests. Both the KO data (*r*_*m*_ = 0.66, *P* = 0.001) and the CAZyme (*r*_*m*_ = 0.49, *P* = 0.004) data were significantly correlated with the molecular composition of DOM. We further explored these relationships between DOM (i.e., FT-ICR MS) and functional potential (i.e., KEGG) using permutational multivariate analysis of variance (PERMANOVA; Supplementary Table 6). Age explained 40% of the variation in overall functional potential of KOs (*P* = 0.008), while DOC concentration explained 19% of KO variation (*P* = 0.041). Although a non-significant trend (*P* = 0.098), NOSC explained 10% of variation in KOs. We further determined that KO distribution explained 42.5% of variation in the FT-ICR MS data (*P* = 0.001), followed by age (24%, *P* = 0.006) and CAZyme distribution (18%, *P* = 0.014).

**FIGURE 5:**
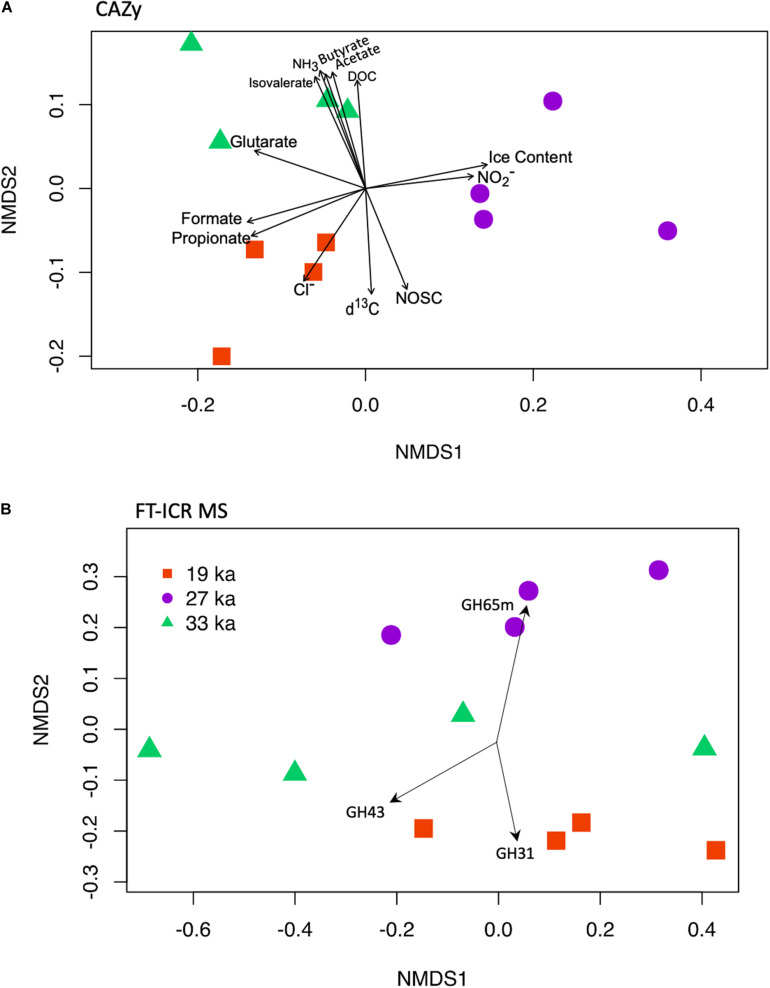
Polysaccharide processing potential and DOC structure differs according to age of samples. Non-metric multidimensional scaling ordination based on Bray-Curtis dissimilarities of **(A)** relative abundance of CAZyme count data with vector fitting of geochemical data (Table 1). **(B)** NMDS based on Bray-Curtis dissimilarities of relative abundance of all identified molecular formulae (FT-ICR MS) with vector fitting of normalized counts of CAZyme involved in polysaccharide degradation (complete list of significantly associated CAZyme PFAMs, with vector coordinates, can be found in Supplementary Table 7). Vectors displayed represent the highest r^2^ associated with each age class.

Ordination of functional gene data with subsequent vector fitting by measured permafrost characteristics indicated that microbial function (Figure 5A and Supplementary Figure 3) was affected primarily by the age of permafrost and associated changes in DOM chemistry. Specifically, distinct differences in the fermentative by-products and permafrost chemistry (e.g., ice content, δ^13^C-DOC, NOSC) were associated with differences in both the functional potential of the microbial community (Supplementary Figure 2) and in CAZyme distribution (Figure 5A). For example, propionate, formate, and chloride concentrations were highest in the youngest (19 ka) samples and lowest in the intermediate samples. Conversely, 27 ka samples had the highest ice content and nitrite concentrations compared to the youngest and oldest. The oldest samples were associated with increases in total DOC, many of the SCFAs (acetate, butyrate, isovalerate), and ammonium. The 33 ka samples were also associated with corresponding decreases in NOSC and lower δ^13^C-DOC (Table 1).

Finally, to investigate the relationship between DOM composition and the potential for carbohydrate processing we ordinated the FT-ICR MS data with vector fitting of the identified CAZymes involved in polysaccharide degradation (Figure 5B). Across samples, 13 GH families were significantly associated with the composition of DOM (Supplementary Table 7). Seven families were associated with DOC composition in the 19 ka samples: potential oligosaccharide-degrading enzymes (GH2, GH3, GH31), potential xylanase (GH10), a hydrolase (GH29, Alpha_L_Fucosidase), and a potential chitobiosidase (GH20) involved in chitin degradation. In the 27 ka samples, three families were associated with the variation in DOM composition: a potential maltose phosphorylase (i.e., GH65m and GH65N domains) and the α-glucoside from GH family 31 and 15 which are associated with starch degradation. Finally, the three CAZyme domains that were associated with the 33 ka DOC composition were a debranching enzyme (GH78), an oligosaccharide-degrading enzyme (GH43), and a potential pectin-degrading enzyme (GH88).

## Discussion

**FIGURE 6:**
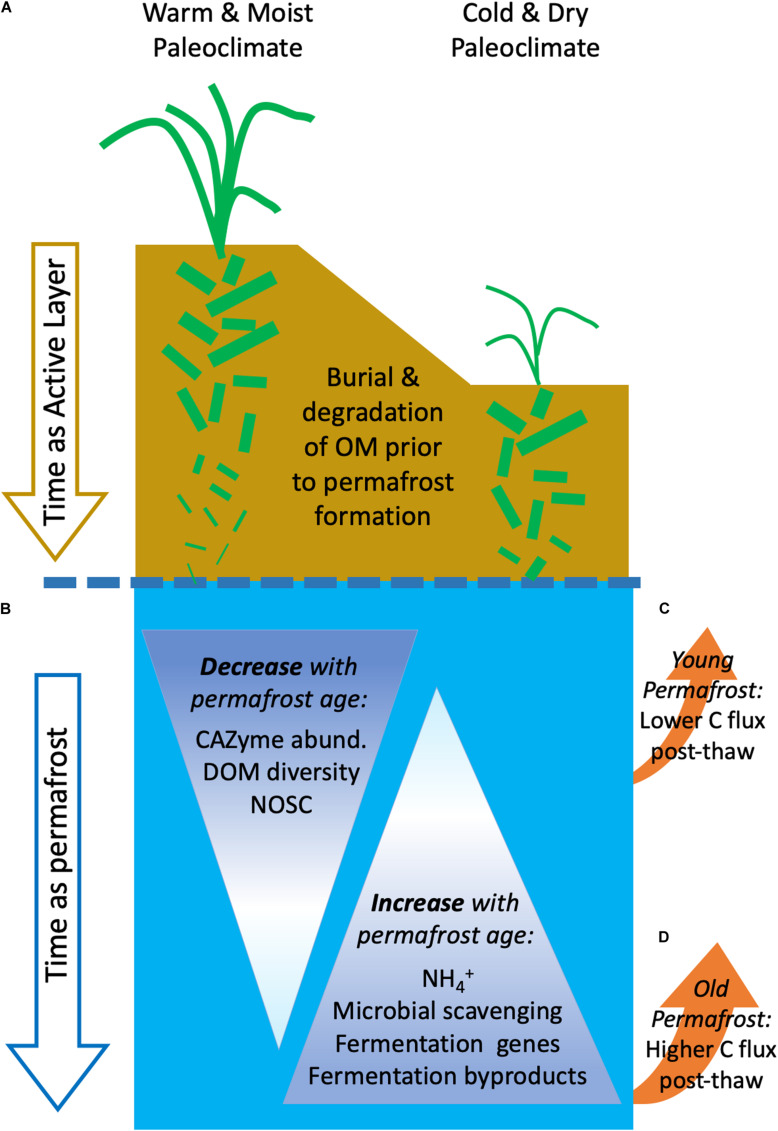
Conceptual diagram of the factors influencing microbial processing of C in permafrost. The dashed blue line indicates the transition from active layer soil to permafrost. The decreasing size of green rectangles represents increasing degree of OM decomposition prior to entrainment in permafrost. **(A)** Under warm and moist paleoclimate conditions, permafrost may have taken centuries to millennia to form (e.g., 19 and 33 ka), meaning that plant-derived C was exposed to microbial processing for longer periods of time (Hamilton et al., 1988). In contrast, when paleoclimate was cold and dry (e.g., 27 ka), permafrost formed in decades to centuries resulting in permafrost SOM which is less microbially processed (Guthrie and Stoker, 1990). **(B)** Once SOM is entrained within permafrost, *in situ* microbial fermentation of buried OM continues slowly across geologic time. Microbial activity slows and stalls as thermodynamic limits are reached. **(C)** As permafrost thaws, younger permafrost contains lower DOC concentrations that is in a chemical form which may slow its input into the global C cycle. **(D)** Because older permafrost contains more biologically active N and SCFAs, thaw may induce rapid metabolism and input of yedoma-C to the atmosphere.

Permafrost contains vast stores of OM and is at risk of increased thaw. Yet the variation in OM chemistry is not well constrained, in part because of our limited understanding of the initial OM entrained in permafrost, but also because of poor recognition that microbial communities in permafrost are themselves altering its chemical nature. Recently, Mackelprang et al. demonstrated that microbial populations adapt and survive in permafrost across the Fox Tunnel chronosequence (Mackelprang et al., 2017; Burkert et al., 2019). These previous studies of the Fox Permafrost Tunnel found that the relative abundance of genes and pathways related to long-term survival increased with age and that vegetative cells persist across the same chronosequence (Mackelprang et al., 2017; Burkert et al., 2019). However, the question of how these microbial populations interact with permafrost OC remains. Here we demonstrate that the molecular composition of DOM changes with permafrost age and we argue these alterations are due in part to shifts in microbial processing of DOM that occurs in permafrost over time (Figures 1–3). We observed multiple strong relationships between community functional potential and the surrounding DOC (Figures 3–5 and Supplementary Figures 2A,B,3). Together, these results describe a complex association between vegetation present during permafrost formation and microbial function over geologic time which interact to affect the ultimate character of permafrost DOM (Figure 6).

The solid and liquid phases of C in permafrost are largely controlled by composition of soil C present, paleoclimate, and vegetation at the time of permafrost formation. At each age along the chronosequence, paleoclimate and paleovegetation likely differed at the time of formation (Figure 6; Jørgensen et al., 2012; Willerslev et al., 2014; Schirrmeister et al., 2016; Mackelprang et al., 2017). The inferred regional paleoclimate, time to permafrost formation, and vegetation composition was more similar between the more mesic 19 and 33 ka than with the colder 27 ka (Hopkins, 1982; Hamilton et al., 1988; Muhs et al., 2001; Mackelprang et al., 2017). Prior to entrainment in permafrost, OM is subject to microbial decomposition. The longer this decomposed material takes to become incorporated into syngenetic permafrost, the more transformation that OM likely undergoes. The relative similarities in paleovegetation and paleoclimate, and therefore the amount of time that OM was available for microbial turnover prior to entrainment within the permafrost, may explain the similarity in DOM, DIN, and ice content between the youngest and oldest ages compared to 27 ka samples (Figure 1A and Table 1). While we observe the effect of these “initial conditions” on permafrost characteristics we also observe significant trends over time indicating that time entrained in permafrost also affects both the microbial community and the molecular composition of DOM (Figures 1, 3–5A, Table 1, and Supplementary Table 3). This is especially true between 19 ka and 33 ka, which were most similar in paleoclimate and vegetation composition but show increases in fermentative products and decreases in DOC degradability, chemodiversity (number of assigned formulae), and secondary fermentative processes across the chronosequence (Figures 1–3, Table 1, and Supplementary Table 5). We therefore posit that while paleoclimate and vegetation affect the initial composition of permafrost C, microbial turnover of that C over time also alters its characteristics (Figure 6). These findings are consistent with those of Mackelprang et al. (2017), as well as a number of single time point studies (Rivkina et al., 2000, 2016; Tuorto et al., 2014; Bottos et al., 2018) that demonstrate while initial conditions at the time of permafrost formation can impact current permafrost communities, over time microbial communities adapt to frozen conditions and microbial metabolism affects the characteristics of dissolved C and N pools. We describe these patterns in more detail below.

### Composition and Degradation of Permafrost Carbon

Permafrost C is comprised of a complex organic mixture including plant debris and structural polymers (Strauss et al., 2015, 2017). Accordingly, we found that plant-derived OM such as lignin- and tannin-like compounds dominated the molecular composition of DOM across the permafrost chronosequence, despite a large reduction in the chemodiversity of DOM over time (Supplementary Table 3). There were few dramatic changes in the relative abundance of broad compound classes of DOM such as lignin or cellulose, despite large difference in chemical composition (Figure 1 and Supplementary Table 3). The most notable changes in permafrost chemistry as it aged were a reduction in the number of identifiable carbon compounds and a decline in NOSC despite overall increases in DOC concentrations (Table 1). Each of these factors suggest *in situ* microbial decomposition of DOC along the chronosequence (Lehmann and Kleber, 2015; Hodgkins et al., 2016) despite frozen conditions.

To understand potential interactions between DOM and the microbial metagenome we screened for genes encoding SCFA analysis (Figure 3) and conserved polysaccharide utilization protein family domains (i.e., CAZymes). We then compared the abundance and diversity of these functional genes to DOC abundance and DOM chemical composition. We found that CAZyme PFAM abundance and diversity decreased with age, which was consistent with fewer unique DOM formulae. Together, these data indicate that as DOM becomes more processed, the microbial community requires a lower diversity of CAZymes to act on that DOM. Because CAZymes have broad substrate specificity we did not directly compare the abundance of any individual functional gene to the abundance of any individual compound class. Instead we relied on multivariate statistical techniques to compare trends in each of the data sets and found microbial functional genes reflected DOC chemistry across the chronosequence. These strong relationships between CAZyme profiles and chemical composition of DOC further indicate that microbial populations respond to the molecular composition and thermodynamic favorability of DOM available.

Cellulose and hemicellulose are the primary plant polymers entrained into terrestrial anaerobic environments, e.g., permafrost (de Souza, 2013). Microbial communities degrade these polymers in a step-wise manner, feeding both themselves and providing products for subsequent microorganisms (Thomson, 1993). Xylan, a major component of permafrost DOM, is a product of cellulose and hemicellulose degradation. Cleavage of ß-1,4-linked D-xylose units is a rate limiting step in this process affected by low temperatures and side-chain substituents (Kotsyurbenko, 2005; de Souza, 2013). In this study, the high relative abundance of xylan-targeting CAZymes in the youngest samples suggests the potential for xylan degradation. The relative abundance of these genes decreased in 33 ka permafrost as the DOM became successively less thermodynamically favorable for carbohydrate degradation (Supplementary Figure 2A). The intermediate aged samples are marked by a high relative abundance of CAZymes associated with both starch and cellulose degradation (Supplementary Figure 2A). This shift could be due a difference in the chemical composition of the soil OM, which formed with an herbaceous-type plant community under colder and drier climate conditions compared with the youngest and oldest permafrost soils (Hopkins, 1982).

Several lines of evidence indicate microorganisms begin scavenging proteinaceous compounds from dead microbial biomass in lieu of macromolecular OM over increasing periods of time since permafrost formation. Mackelprang et al. (2017) used metagenomics to suggest microorganisms shift from plant compounds to proteinaceous compounds over time. Ewing et al. (2015a,b) showed increasing concentrations of DOC downcore (i.e., with increasing age) through permafrost up to 134 ka and associated increases in ammonium that could have only been produced *in situ*. In this study we observe an increase in CAZymes associated with peptidoglycan degradation in the oldest permafrost (Polysaccharide deacetylase and lysin motifs; Supplementary Figure 2A). This suggests degradation of bacterial cell walls, which is further evidence for microbial scavenging of detrital biomass as energy rich OM becomes limiting and imparts severe limitations on microbial growth. As microorganisms deplete thermodynamically favorable electron donors and acceptors, those that persist may need to flexibly scavenge available C and N (Bottos et al., 2018; Burkert et al., 2019).

As microbes scavenge for available C, products of anaerobic OM hydrolysis can serve as substrates for microbial fermentation: a common microbial process found in Alaskan permafrost metagenomes (Lipson et al., 2013; Mackelprang et al., 2016). Acetate, formate, and other SCFAs are central intermediates in the fermentation of OM, and precursors to CH_4_ and CO_2_ formation (Hädrich et al., 2012; Ye et al., 2014; Ewing et al., 2015a). Increasing SCFA concentration along with key fermentation genes (Figure 3) across the chronosequence indicates anaerobic decomposition via fermentation as has been observed previously in Interior Alaskan yedoma (Drake et al., 2015; Ewing et al., 2015a). Dictated by thermodynamics, fermentation characteristically occurs under strongly reducing conditions (e.g., CO_2_, sulfate) where alternative terminal electron acceptors (e.g., nitrate, nitrite, sulfate, FeIII) have been depleted. The oxidation state of OM itself can also indicate the extent to which it is decomposed (Boye et al., 2017, 2018). An abundance of low (more negative) NOSC C compounds may favor less thermodynamically favorable microbial processes such as fermentation and acetogenesis (Ye et al., 2014; Ewing et al., 2015a; Boye et al., 2018). Our data indicate increased pooling of reduced C and SCFAs in the oldest samples (Figure 2), suggesting that less thermodynamically favorable C is preserved in ancient permafrost.

The increasing concentrations of electron donors such as DOC and NH_3_ with increasing permafrost age are another indication of anaerobic decomposition via fermentation (Duddleston et al., 2002; Hädrich et al., 2012; Ewing et al., 2015a,b). However, there should be a net accumulation of organic acids in anaerobic sediments when rates of fermentation exceed rates of respiration and methanogenesis. Under extremely low redox conditions anaerobic metabolism that includes respiration, methanogenesis, and fermentation will slow if inhibiting end products such as SCFAs are present (Jackson and McInerney, 2002). Our data show pooling of these SCFAs indicating a stall in primary and secondary fermentative processes in older permafrost and that the associated microbial populations may be reaching the thermodynamic limits of anaerobic metabolism (Figures 2, 3 and Table 1). Indeed, the anaerobic metabolism of isovalerate is dependent on syntrophic fermentative microbial interactions (Stieb and Schink, 1986), and we observe a significant pooling of isovalerate with increasing permafrost age (Table 1).

Our research indicates both the initial conditions of permafrost soils and microbial activity over time can affect the quantity and chemistry of DOM in permafrost soils. Abiotic factors such as potential mineral dissolution, composition of OM prior to freezing, and other physical processes also play a role in the shaping of permafrost DOM pools (Drake et al., 2015; Ewing et al., 2015b). In the future context of permafrost thaw, differences in DOC chemistry released after thaw could affect soil and aquatic biogeochemistry, as DOC quantity and DOM chemical characteristics impact soil and aquatic CO_2_ fluxes (Drake et al., 2015; O’Donnell et al., 2016). The pooling of biolabile organic acids and biologically available N in older permafrost could prime the system for a larger and more rapid microbial response in contrast to younger permafrost soils (Figure 6).

Knowledge of the factors controlling microbial survival and the molecular composition of OM in Pleistocene-aged permafrost is scarce. Through this work, we show further evidence for microbial survival across millennia and that those microbial populations may be actively modifying permafrost DOM *in situ*. Here we examined three time points from the late Pleistocene, which may not be representative of other paleoclimate or permafrost conditions. To improve our understanding of microbial activity of the cryosphere across geologic timescales, further research should incorporate other permafrost chronosequences with similar parent material and paleovegetation, as well as age ranges beyond those examined here.

Finally, further exploration of microbial mechanisms of survival in extreme environments could inform the astrobiological search for life on other planets, as permafrost on Earth may be a representative model of extraterrestrial icy habitats, such as Mars, Enceladus, or Europa (Rivkina et al., 2010). Our work highlights the fact that soil microorganisms are active in relatively warm permafrost (−3°C) and that functional gene abundance is indicative of changing soil microbial processes. Further exobiology work could use exploration of functional genes in combination with stable isotope probing or other methods to determine the changing patterns of microbial function in exoplanet permafrost analogs.

## Data Availability Statement

The datasets generated for this study can be found in the NCBI short read archive accession number SRP093781, all chemistry, FT-ICR MS, and CAZyme data can be found in the USGS Science Base Data Repository at: https://www.sciencebase.gov/catalog/item/5cd08fb1e4b09b8c0b79a466 and https://doi.org/10.5066/P933APLH.

## Author Contributions

M-CL statistically analyzed all data and wrote the original draft manuscript. RM performed the metagenome analyses. RB and AS performed the CAZyme analyses. RB contributed to the statistical analysis and interpretation of CAZyme data. DP, PZ, and RS performed the FT-ICR MS analyses and data processing. CC and JM performed the permafrost filtrate analyses. MW and JM assisted with field work. MW and RM provided the funding. TD provided access to the field site and oversaw sample coring. All authors reviewed and contributed to the final version of the manuscript.

## Conflict of Interest

The authors declare that the research was conducted in the absence of any commercial or financial relationships that could be construed as a potential conflict of interest.

## Footnotes

1 http://hannonlab.cshl.edu/fastx_toolkit/

## Supplementary Material

FIGURE S1 **(A)** Photograph taken by M.C. Leewis from the entrance to the CRREL Fox Permafrost Tunnel looking back toward three sampling sites. **(B)** Cross section of the CRREL Fox Permafrost Tunnel identifying general lithology and approximate location of each core collected (circles). The age of expose permafrost increases inward from the tunnel portal. Figure adapted from Mackelprang et al. (2017) and Burkert et al. (2019), and **(B)** redrawn from Hamilton et al. (1988).

FIGURE S2 Heatmap of CAZymes significantly representing **(A)** functions affected by age (*P* < 0.01) or **(B)** functions stable across the tested ages (*P* > 0.01). Colors represent the correlation with age, with warmer colors representing a stronger correlation between CAZyme and age. Abundances are scaled by row.

FIGURE S3 Non-metric multidimensional scaling (NMDS) plot representing the Bray Curtis dissimilarity of all identified KO’s in the permafrost metagenome from across all sites with subsequent vectors of environmental and geochemical data onto the ordination (*P* < 0.05).

TABLE S1 Characteristics of compound classes used to categorize FT-ICR MS molecular formulae. Criteria based on Hodgkins et al. (2016).

TABLE S2 List of protein families (Pfam ID), with corresponding domain names, of potentially involved in polysaccharide processing (CAZymes) identified in metagenome data. The indicated *P-*value corresponds to one-way ANOVA test on CAZyme abundance in each age class to determine those which were affected (*P* < 0.01) or unaffected (*P* > 0.01) by age. The indicated columns represent the mean and standard deviation (SD) CAZyme abundance for: all (All), 19ka, 27 ka, or 33 ka samples.

TABLE S3 FT-ICR MS results representing the distribution of DOC leachate by compound class from the Fox Tunnel Permafrost Chronosequence. Data other than total number of formulae and molecular weight (MW) are the average relative abundance of the three replicates with standard deviation from the mean. Compound classes defined in Supplementary Table 1. Abundance weighted average MW calculated as per Hodgkins et al. (2016).

TABLE S4 Multiple-response permutation procedure (MRPP) tests for differentiation of molecular composition of DOC, KO gene counts, and CAZyme Pfam counts.

TABLE S5 Relative abundances of KEGG genes in metabolic pathways described in Supplementary Figure 3. Significance of age class comparisons are denoted.

TABLE S6 Permutational multivariate analysis of variance (PERMANOVA) table exploring factors which may explain variation in either the molecular composition of DOC (FT-ICR MS) or functional potential (KEGG).

TABLE S7 CAZyme domains which are significant vectors explaining the FT-ICR MS data.
