# Supplementary material for: Life at the Frozen Limit: Microbial Carbon Metabolism Across a Late Pleistocene Permafrost Chronosequence
Source: Front Microbiol. 2020 Jul 29;11:1753. doi: 10.3389/fmicb.2020.01753 (PMC7403407; doi:10.3389/fmicb.2020.01753)
Supplement: FIGURE S1 — (A) Photograph taken by M.C. Leewis from the entrance to the CRREL Fox Permafrost Tunnel looking back toward three sampling sites. (B) Cross section of the CRREL Fox Permafrost Tunnel identifying general lithology and approximate location of each core collected (circles). The age of expose permafrost increases inward from the tunnel portal. Figure adapted from Mackelprang et al. (2017) and Burkert et al. (2019), and (B) redrawn from Hamilton et al. (1988). [file Data_Sheet_1.docx]

**Supplementary Figures**

**Supplemental Figure 1:** A) Photograph taken by M.C. Leewis from the entrance to the CRREL Fox Permafrost Tunnel looking back towards three sampling sites. B) Cross section of the CRREL Fox Permafrost Tunnel identifying general lithology and approximate location of each core collected (circles). The age of expose permafrost increases inward from the tunnel portal. Figure adapted from (Mackelprang *et al.*, 2017; Burkert *et al.*, 2019), and panel B redrawn from (Hamilton *et al.*, 1988).

**Supplemental Figure 2.** Heatmap of CAZymes significantly representing **(A)** fluctuating functions (*P* < 0.01) or **(B)** stable functions (P > 0.01) across the tested ages. Colors represent the correlation with age, with warmer colors representing a stronger correlation between CAZyme and age. Abundances are scaled by row.

**Supplementary Figure 3.** Non-metric multidimensional scaling (NMDS) plot representing the Bray Curtis dissimilarity of all identified KO’s in the permafrost metagenome from across all sites with subsequent vectors of environmental and geochemical data onto the ordination (*P* < 0.05). Abbreviations are as follows: A is acetate, B is butyrate, I is isovalerate, P is propionate, F is formate, G is glutarate, M is malic acid.
